# Usefulness of Approximate Entropy in the Diagnosis of Schizophrenia

**Published:** 2011

**Authors:** Mahsa Taghavi, Reza Boostani, Malihe Sabeti, Seyed Mohammad Arash Taghavi

**Affiliations:** 1 Department of Psychiatry and Behavioral Sciences, Shiraz University of Medical Sciences, Shiraz Iran; 2 Department of CSE&IT, Faculty of Electrical and Computer Engineering, Shiraz University, Shiraz, Iran.; 3 Isfahan University of Medical Sciences.

**Keywords:** ApEn, EEG, Schizophrenia, SVM

## Abstract

**Objectives:** Diagnosis of the psychiatric diseases is a bit challenging at the first interview due to this fact that qualitative criteria are not as accurate as quantitative ones. Here, the objective is to classify schizophrenic patients from the healthy subject using a quantitative index elicited from their electroencephalogram (EEG) signals.

**Methods**: Ten right handed male patients with schizophrenia who had just auditory hallucination and did not have any other psychotic features and ten age-matched right handed normal male control participants participated in this study. The patients used haloperidol to minimize the drug-related affection on their EEG signals. Electrophysiological data were recorded using a Neuroscan 24 Channel Synamps system, with a signal gain equal to 75K (150 xs at the headbox). According to the observable anatomical differences in the brain of schizophrenic patients from controls, several discriminative features including AR coefficients, band power, fractal dimension, and approximation entropy (ApEn) were chosen to extract quantitative values from the EEG signals.

**Results:** The extracted features were applied to support vector machine (SVM) classifier that produced 88.40% accuracy for distinguishing the two groups. Incidentally, ApEn produces more discriminative information compare to the other features.

**Conclusion: **This research presents a reliable quantitative approach to distinguish the control subjects from the schizophrenic patients. Moreover, other representative features are implemented but ApEn produces higher performance due to complex and irregular nature of EEG signals.

## Introduction

Schizophrenia is a severe and persistent debilitating psychiatric disorder. Diagnosis of schizophrenic patients is mostly performed based on qualitative criteria. According to the diagnostic criteria of the American Psychiatric Association (DSM-IV) (1), patients show disturbances in thoughts (or cognitions), affects, and perceptions and difficulties in relationships with others. In schizophrenia, a major enduring split exists between affect and thoughts. The hallmark symptoms of schizophrenia are the experiences of hallucinations, often of the auditory type, as well as delusions. 

Electroencephalogram (EEG) has been an important clinical tool for the evaluation and diagnosis of brain diseases. First attempts to apply methods from nonlinear time series analysis to EEG were carried out in the framework of the chaos hypothesis. It was assumed that the EEG within a particular psycho-physiological state could be described by a deterministic chaotic system and therefore could be characterized by invariant measures such as the fractal dimension or as Lyapunov exponents. Recently, much attention is given to analysis of EEG signals of schizophrenic patients (2) Lee et al. ‎3 detected the non-linearity in the schizophrenia with a modified method of surrogate data. They showed the correlation dimension could be used as a discriminating statistic to demonstrate non-linearity in the EEG. Jeong et al. (4) stated that the value of D_2 _in the left inferior frontal and anterior temporal regions in 13 schizophrenic patients is decreased compared to eight healthy controls. Kim et al (5) reported decreasing of first-Lyapunov exponent in the frontal regions of 25 schizophrenic patients in comparison with 15 healthy controls. 

The disturbances of the normal sleep EEG architecture associated with schizophrenia were also investigated from a nonlinear perspective. Kirsch et al. (6) reported that during the performance of a cognitive task, the D2 of healthy patients’ EEG decreased. This change did not occur in patients with schizophrenia performing the same task. Sabeti et al. (7) selected the best frequency bands by genetic algorithm to classify the schizophrenic and control participants. 

Approximate entropy (ApEn) is another parameter recently introduced to quantify regularity in data without any prior knowledge about the system generating them. It was constructed by Pincus, motivated by applications to short and noisy data sets, along thematically similar lines to K-S entropy ‎7. However, the focus was, in this case, to provide a widely applicable, statistically valid formula that will distinguish data sets by a measure of regularity. The observation motivating ApEn is that if joint probability measures of reconstructed dynamics that describe each of two systems are different, then their marginal probability distributions on a fixed partition, given by conditional probability, are likely different. Typically, orders of magnitude fewer points are needed to accurately estimate these marginal probabilities than to accurately reconstruct the attractor measure defining the process. 

Based on numerous studies, ApEn may correlate with hidden changes often undetected by other more classical time series analyses including spectral analysis and correlation dimension. ApEn changes have often been seen to be predictive of subsequent clinical changes. This has facilitated the application of ApEn to numerous settings both within and outside of biology. Preliminary evidence suggests that ApEn of EEG is predictive of epileptic seizures ‎9 . It is also applied to extract features from EEG and respiratory recordings of a patient during Cheyne-Stokes respiration (10) and to quantify the depth of anesthesia  ‎14. 

The objective of this study is to evaluate the estimated ApEn of schizophrenic patients’ EEGs compared to healthy subjects. The reminder of this paper is organized as follow: Section 2 explains the experimental setup, the task and the basic data preprocessing. Section 3 introduces the employed features and section 4 briefly describes the SVM (12) classifier. Experimental results are given in section 5. Finally a discussion and conclusion part is presented. 

## Materials and Methods

*Data acquisition*

Ten patients with schizophrenia and ten age-matched control participants (all male, uniformly distributed in the interval of 18-55 years old) participated in this study. They were recruited from the Center for Clinical Research in Neuropsychiatry, Perth, Western Australia. According to DSM-IV criteria (1), the patients were diagnosed as having a lifetime schizophrenia or schizophrenia spectrum disorder. The patients were not divided in some sub-groups regarding sub-type of schizophrenia. The patients used haloperidol to minimize the drug-related affection on their EEG signals. It should be noted that the history reports of both groups confirmed that normal participants did not have any psychotic symptom and also our patients had just auditory hallucination and did not have any other psychotic features. 

**Figure 1:**
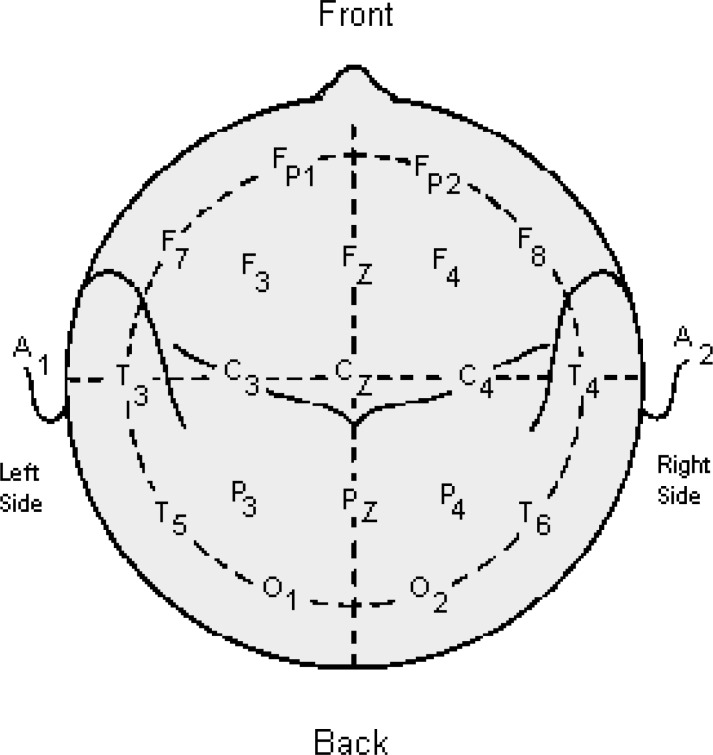
The head partition and electrodes positioning

The signals were recorded when the patients were in the remission phase, otherwise the signal recording could not be performed. Each participant was seated upright with eyes open and the experiment lasted for two minutes. Electrophysiological data were recorded using a Neuroscan 24 Channel Synamps system, with a signal gain equal to 75K (150 xs at the headbox). For EEG paradigms, 20 electrodes (Electrocap 10-20 standard system (13) were recorded plus left and right mastoids, VEOG (14) and HEOG (14). In the EEG paradigms, eye-blink artifacts were corrected using the technique proposed in ‎15, and manually screened for artifact. EEG data were recorded from 20 electrodes (Fpz, Fz, Cz, Pz, C_3_, T_3_, C_4_, T_4_, Fp_1_, Fp_2_, F_3_, F_4_, F_7_, F_8_, P_3_, P_4_, T_5_, T_6_, O_1_, O_2_) with sampling frequency rate at 200 Hz. Figure 1 shows the head partition and electrodes positioning.

*Approximate Entropy*

ApEn was introduced as a quantification of regularity in sequences and time series data, initially motivated by applications to relatively short, noisy data sets. Mathematically it is part of a general development of approximating Markov Chains to a process. It provides a finite sequence formulation of randomness, via proximity to maximal irregularity. A statistical evaluation of ApEn is available in. ApEn is a scale invariant feature which reveals both dominant and subordinant (17) information within a time frame. Therefore, ApEn is repeatedly considered as an informative feature that is led to highly discriminate EEG signals of similar diseases (17). Notably it detects changes in underlying episodic behavior not reflected in peak occurrences or amplitudes. It is applicable to systems with least 50 data points and to broad classes of models; it can be applied to discriminate both general classes of correlated stochastic processes, as well as noisy deterministic systems. 

Moreover, ApEn is complementary to spectral and autocorrelation analyzes, providing effective discriminatory capability instances in which the aforementioned measures exhibit minimal distinctions. It is nearly unaffected by low level noise, is also robust to meaningful information with a reasonable number of data points, and is finite for both stochastic and deterministic processes. It measures the logarithmic likelihood that runs of patterns that are close remain close on subsequent incremental comparisons, and assigns a nonnegative number to a time series, with larger values corresponding more complexity or irregularity in the data. ApEn has two user-specified parameters: a run length m and a tolerance window r. It is important to consider ApEn (m, r) or ApEn (m, r, N), where N is the number of points of the time. 

Formally, given   data points from a time series {*x(n)=x(1),x(2), …, x(N)*, to compute ApEn, one should follow these steps.

- Form *m*-vectors *X(1), …,X(N-m+1)* defined by:

$x\left(i\right),x\left(i+1\right),\ldots ,x(i+m-1)],$

i=,…N-m+1                                  (1)

These vectors represent  consecutive  values, commencing with the  th point.

- Define the distance between *x(i)* and *X(j)*, *d[X(i), X(j)]*, as the maximum absolute difference between their respective scalar components, i.e., the maximum norm

$d\left[X\left(i\right),X\left(j\right)\right]=\underset{k=1,2,\ldots ,m}{\max}|x\left(i+k-1\right)-x(j+k-1)$    (2)

- For a given *X(i)*, count the number of *j)j=1,…,N-m+1,j#i)* so that *d*[x(i),X(j*)*]*≤r* , denoted as N^m^ *(i)*. Then, for *i=1,…,N-m=1*

c(i)rm=Nm(i)(N-m+1)           (3)

c(i)rm measures, within a tolerance *r* , the frequency of patterns similar to a given one of window length *m*.

- Compute the natural logarithm of each c(i)rm, and average it over *i*

∅mr=1N-m+1∑i=1N-m+1lnc(i)rm          (4)

- Increase the dimension to *m*+1  . Repeat steps 1-4 and find c(i)rm and $\emptyset^{m+1}\left(r\right)$.
- ApEn is defined as

$\mathrm{ApEn}\left(m,r,N\right)=\emptyset^{m}\left(r\right)-\emptyset^{m+1}(r)$          (5)

For the study discussed in this paper, ApEn is estimated using the widely established parameter values of *m*=2, and *r*=0.1times the standard deviation (SD) of the original data sequence. 

*Auto-regressive (AR) coefficients*

AR model is a powerful tool for signal modeling. In this model, each sample is considered as a prediction of previous weighted samples. The number of weights determines the model order. Here, autoregressive coefficients are estimated by Burg method (19). The Burg method fits an AR model (order P), which is shown in the equation (6), to the input signal x. The process of signal modeling is performed by minimizing the forward and backward prediction errors while constraining the AR coefficients, $\alpha_{i}$, to satisfy the Levinson-Durbin recursion. 

$x\left(t\right)=-\sum_{i=1}^{P}a_{i}x(t-i)$ *(6)*

*Band Power*

EEG contains different specific frequency components which some of them carry the discriminative information. This feature reflects the energy of alpha, beta, theta and delta bands which are particularly important to classify the different brain states. At first, EEG signals have been filtered by four Butterworth band pass filters (order five) in 8-13 Hz (alpha band), 13-30 Hz (beta band), 4-8 Hz (theta band) and 0-4 Hz (delta band). Then, the filtered signals are squared to determine the signal power in each windowed signal. 

*Fractal dimension*

Fractal dimension (20) has a direct relation with the amount of information inside a signal, and can be interpreted as the degree meandering (or roughness or irregularity) of a signal. Consider x (1), x (2), x (N) the time sequence to be analyzed. Construct k time series $x_{m}^{k}$ as follow:

$x_{m}^{k}=\{x\left(m\right),x\left(m+k\right),x\left(m+2k\right),\ldots x\left(m+\left|\frac{N-m}{k}\right|k\right)\}$          (7) 

where m=1, 2, …, k, m shows the initial time and k shows delay between points. For each time series $x_{m}^{k}$, the average length $L_{m}(k)$ is computed as:

$L_{m}\left(k\right)=\frac{\left(N-1\right)\left|\frac{N-m}{\sum_{i=1}^{k}}\right|x\left(m+ik\right)-x(m+\left(i-1\right)k}{\left|\frac{N-m}{k}\right|k}$           (8)

where N is the length of time sequence. Total average length *L(k)* is computed for all time series having the same delay k but different m as: 

$L\left(k\right)=\sum_{m=1}^{k}L_{m}(k)$          (9)

This procedure is repeated for each k ranging from 1 to *k*_max_, the total average length for delay k, L (k), is proportional to *k*^-D^, where D is the fractal dimension by Higuchi's method. In the curve of ln(L(k)) versus ln(1/k), the slope of the least-squares linear best fit, is the estimate of the fractal dimension. 

*Classifier*

The main idea of SVM (21) is to construct a hyper-plane as a decision surface in such a way that the margin of separation between positive and negative examples is maximized. The support vector machine is an approximate implementation of the method of structural risk minimization. The SVM, given labeled training data

$D=\{\left(x_{i},y_{i}\right)\}l_{i=1}$  

x$x_{i}\in X\subset R^{d},y_{i}\in Y=\{-1,+1\}$           (10)

constructs a maximal margin linear classifier in a high dimensional feature space ∅(x) defined by a positive definite kernel function $k(x,x^{'})$ specifying an inner product in the feature space, 

$\emptyset \left(x\right)\mathrm{.\emptyset}\left(x^{'}\right)=k(x,x^{'})$            (11)

A common kernel is the Gaussian radial basis function (RBF), 

$k\left(x,x^{'}\right)=e^{{-\left\|x-\mathrm{x'}\right\|}^{2}}/{2\sigma }^{2}$           (12)

The function implemented by a support vector machine is given by

$f\left(x\right)=\left\{\sum_{i=1}^{l}a_{i}y_{i}k\left(x_{i},x\right)\right\}-b$            (13)

To find the optimal coefficients  of this expansion, it is sufficient to maximize the function, 

$W\left(\alpha \right)=\sum_{i=1}^{l}a_{i}-\frac{1}{2}\sum_{i,j=1}^{l}y_{i}y_{j}a_{i}\alpha_{j}K\left(x_{i},x_{j}\right)$          (14)

## Results

**Figure 2:**
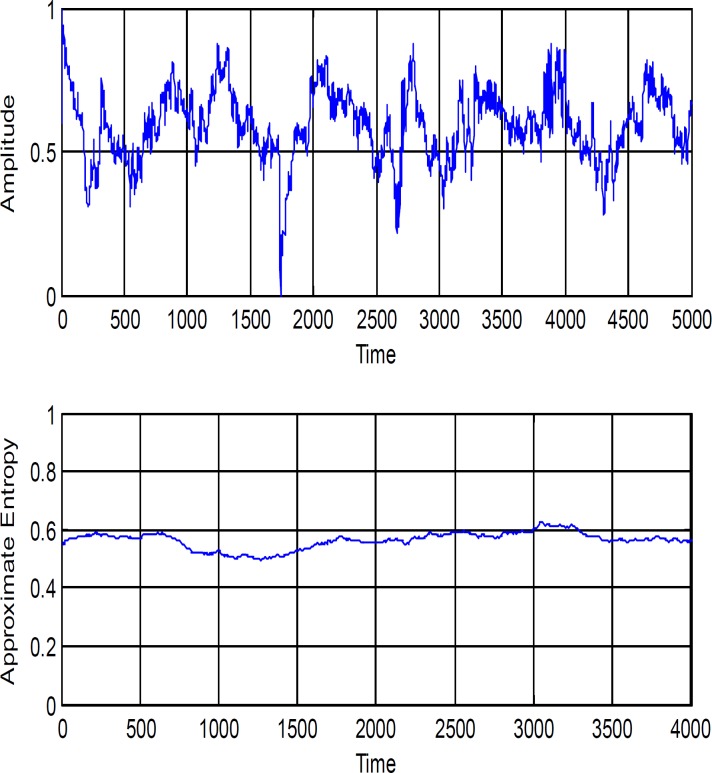
A typical EEG signal of a schizophrenic patient in the time domain along with its ApEn changes is showed above

In order to study the difference between ApEn of healthy and schizophrenic participants, the ApEn is extracted from successive windowed signals that each takes 2 seconds and successive frames have 50% overlap. Resulting time series was constructed from ApEn values calculated within windows sliding in one steps. A trial of our EEG dataset along with its ApEn index is shown in Figure 2. 

For the other features, the EEG signal is divided into the same window size to fairly validate the assumption of stationary. For each windowed signal, we have extracted AR coefficients, band power and Higuchi fractal dimension. After extraction of the features, the estimated ApEn, AR coefficients, band power and Higuchi fractal dimension for five channels are used as inputs to SVM classifier. The important point in the validation phase is that to avoid having a correlation between the train and test feature vectors, each time, feature vectors of each participant is considered as test and the rest is considered as train set. Here, we call it leave-one (participant)-out cross validation method. 

Tables 1-5 show mean ± standard deviation of ApEn for Cz, C3, C4, T3 and T4. These channels are studied because they are located in the temporal lobes located over the limbic area. The neuro-psychological findings state the difference between the EEG indexes of schizophrenic and normal participants is more highlighted in this area (20). 

**Table 1:** Estimated ApEn for Cz channel

Cz	Schizophrenic	Healthy
1	0.7205 ± 0.0512	0.5636 ± 0.0286
2	0.6656 ± 0.0610	0.6723 ± 0.0292
3	0.6220± 0.0301	0.6050 ± 0.0287
4	0.6336 ± 0.0295	0.4244 ± 0.0184
5	0.5057 ± 0.0467	0.4686 ± 0.0459
6	0.6792 ± 0.0289	0.5709 ± 0.0334
7	0.7048 ± 0.0336	0.7534 ± 0.0416
8	0.5662 ± 0.0297	0.7294 ± 0.0289
9	0.3357 ± 0.0511	0.5996 ± 0.0252
10	0.5155 ± 0.0133	0.4629 ± 0.0401

**Table 2:** Estimated ApEn for C3 channel

C3	Schizophrenic	Healthy
1	0.7158 ± 0.0332	0.6475 ± 0.0309
2	0.7040 ± 0.0421	0.6922 ± 0.0354
3	0.6068± 0.0382	0.5505 ± 0.0273
4	0.6273 ± 0.0160	0.4298 ± 0.0269
5	0.4932 ± 0.0934	0.5196 ± 0.0788
6	0.6669 ± 0.0215	0.6008 ± 0.0351
7	0.5157 ± 0.0352	0.7703 ± 0.0474
8	0.6588 ± 0.0246	0.6956 ± 0.0202
9	0.4773 ± 0.0757	0.6345 ± 0.0199
10	0.3870 ± 0.0554	0.5265 ± 0.0390

**Table 3:** Estimated ApEn for C4 channel

C4	Schizophrenic	Healthy
1	0.4942 ± 0.0324	0.5407 ± 0.0216
2	0.4632 ± 0.0470	0.5791 ± 0.0175
3	0.6655 ± 0.0281	0.6764 ± 0.0230
4	0.5119 ± 0.0208	0.5928 ± 0.0569
5	0.4688 ± 0.0601	0.6278 ± 0.0818
6	0.5075 ± 0.0152	0.5766 ± 0.0590
7	0.6540 ± 0.0377	0.7484 ± 0.0476
8	0.3144 ± 0.0297	0.4418 ± 0.0236
9	0.3672 ± 0.0543	0.4679 ± 0.0453
10	0.3599 ± 0.0164	0.5755 ± 0.0370

**Table 4:** Estimated ApEn for T3 channel

T3	Schizophrenic	Healthy
1	0.6270 ± 0.0333	0.5402 ± 0.0411
2	0.6855 ± 0.0258	0.6801 ± 0.0373
3	0.6049 ± 0.0224	0.6652 ± 0.0442
4	0.6280 ± 0.0219	0.4506 ± 0.0234
5	0.4088 ± 0.0649	0.5879 ± 0.0719
6	0.6389 ± 0.0216	0.5704 ± 0.0464
7	0.6180 ± 0.0593	0.6951 ± 0.0344
8	0.5826 ± 0.0267	0.7045 ± 0.0342
9	0.5498 ± 0.0839	0.5903 ± 0.0249
10	0.3257 ± 0.0140	0.5467 ± 0.0317

**Table 5:** Estimated ApEn for T4 channel

T4	Schizophrenic	Healthy
1	0.6742 ± 0.0606	0.5945 ± 0.0615
2	0.6586 ± 0.0420	0.6278 ± 0.0307
3	0.7614 ± 0.0316	0.6471 ± 0.0326
4	0.6274 ± 0.0159	0.6778 ± 0.0616
5	0.3589 ± 0.0478	0.4772 ± 0.0962
6	0.5921 ± 0.0579	0.6235 ± 0.0266
7	0.6548 ± 0.0361	0.6901 ± 0.0289
8	0.4081 ± 0.0292	0.7226 ± 0.0249
9	0.4707 ± 0.1404	0.3590 ± 0.0384
10	0.3597 ± 0.0173	0.5286 ± 0.0323

The classification accuracy using leave-one (participant)-out cross validation by considering the features of the mentioned channels is shown in table 6. To demonstrate statistical significance of the achieved results, F-test and pair T-test were applied on the classification results. All calculated F-test values were higher than 1 and the P-values determined less than 0.05 that confirms the significant supremacy of ApEn compare to the other features. 

In order to analyze whether the performance of each feature is biased to one of the two groups or not, sensitivity (true positive ratio) and specificity (true negative ratio) of the results are calculated by the following statistical indexes: 

$\mathrm{sensitivity}=\frac{\mathrm{TP}}{\mathrm{TP}+\mathrm{FN}},\mathrm{specificity}=\frac{\mathrm{TN}}{\mathrm{TN}+\mathrm{FP}}$

$\mathrm{and Accuracy}=\frac{\mathrm{TP}+\mathrm{TN}}{\mathrm{TP}+\mathrm{TN}+\mathrm{FP}+\mathrm{FN}}$

where TP=true positive; TN = true negative; FP = false positive; and FN = false negative. The sensitivity and specificity of the results for all features were led to the similar values due to equal population of patients and control subjects. 

**Figure 3:**
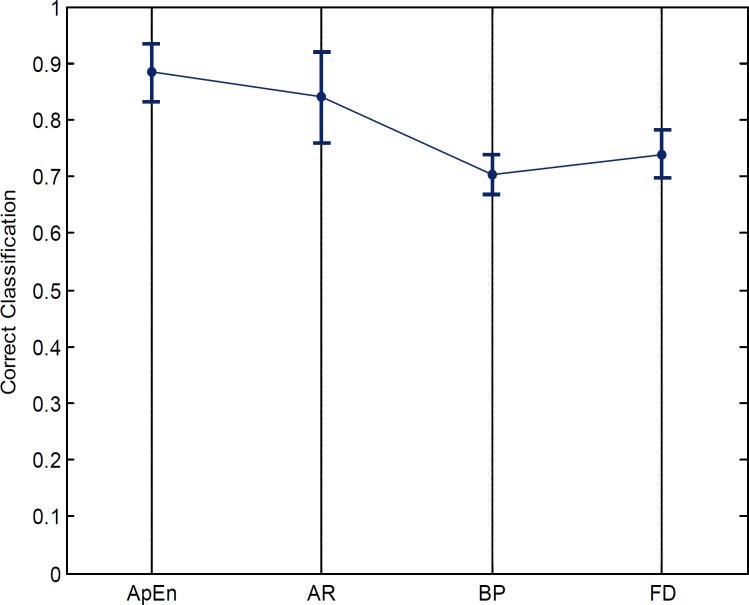
Test results of SVM (Mean ± Std) using different features [ApEn, AR, Band Power (BP), Fractal Dimension (FD)]

In figure 3 the classification accuracy of the employed features are depicted. It is shown the ApEn is more informative than the other features for classifying the two groups. 

**Table 6:** Classification accuracy using leave-one (participant)-out cross validation method.

Feature Type	Accuracy (Mean ± Std)
ApEn	0.8840 ± 0.0509
AR	0.8401 ± 0.0801
Band Power	0.7040 ± 0.0353
Fractal Dimension	0.7390 ± 0.0430

## Discussion

As far as schizophrenic patients thoughts are not complex and in the arbitrary tasks, these patients tend to be repetitive rather using a vast variety of choices (22), it is expected to achieve lower complexity value in their EEG signals. Hence, the signals entropy (which is related with the amount of chaotic behavior of a signal) is employed here to represent the complexity values in the two mentioned groups. In this study, a fast method denotes as ApEn is employed to extract the entropy of EEG signals and also SVM classifier is applied to the extracted features for distinguishing the two groups. 

The extracted complexity values for normal subjects were remarkably higher than that of schizophrenic patients. These changes are significantly highlighted in those channels located over the limbic area of the brain. The anatomical and functional changes in the limbic systems of schizophrenic patients compare to that of healthy subjects have been observed in fMRI and PET images that are vastly reported in the literatures (23,24) Hence, only the recorded EEGs from the Cz, C3, C4, T3 and T4 channels were analyzed to avoid the redundancy. 

In similar studies, the compared features such as band power (25), fractal dimension (26, 27), and AR coefficients (28) were considered as discriminative features to classify psychotic patients from controls. Most of these studies use auditory stimulus to find a difference in response of their (evoke potential) to this external inputs. Although some of these attempts lead to exhibit significant results, none of them apply their methods to raw EEG signals. This reason is that analyzing the raw EEG is much harder rather focusing on just differences in auditory evoke potential (AEP). 

For example, band power feature is very discriminative when an imagery movement (similar to the brain computer interface application) is requested from the subject in the recording protocol; otherwise, no physiological fact exists to change the discharge rate of neurons in different brain lobe at the restful condition. 

AR coefficients try to model the time or spectral behavior of a signal trial. Although EEG signals behave noisy and it is assumed spectrum of such signal should significantly varies, as far as the brain state does not change, the frequency content of this noisy signal does not remarkably varying. Therefore, we do not expect to see a dramatic change in the AR coefficients between the normal and schizophrenic subjects. 

Due to the irregular behavior of EEG signals, fractal dimension and entropy (complexity) based features seem being informative. If our application was an offline process and we accessed to large number of samples, the results of fractal dimension and ApEn would be fairly similar. Complexity and entropy based features are closely related to each other such that entropy of a signal is related with the complexity and fractal dimension of that signal (29,30,31) Moreover, ApEn estimated entropy of a signal much faster than the state-of-art methods of computing the fractal dimension such as correlation dimension, Higuchi, Hurst exponents or dominant Lyapunov exponent methods. 

As it can be seen in table 6, ApEn provided a more precise result because the window length is limited and ApEn does not need large number of samples to produce a reliable index, while performance of fractal dimension is highly dependent of the length of the signal (number of samples). In addition, ApEn index for a short length signal is very fast to compute and is efficient for online decision making process. 

Leave-one (participant)-out cross validation method is applied to our experimental data to minimize the over-fitting affect by removing the correlation between train and test sets. Finally the results with the ApEn show 88.40% accuracy between the two groups that significantly outperformed the rival features (Fig. 3). 

Another advantage of the proposed approach is that without using the beamforming or localization methods, we can find out the key areas in which maximum changes is occurred between the two groups. In other words, if we consider the features of all 20 channels, not only no improvement would be achieved but also the classification rate would be decreased due to increasing the redundant features leading to incline the complexity while feed no more information to the features of the mentioned channels. 

SVM is a power classifier which simultaneously minimizes the structural risk while maximizing the classification accuracy. Unlike other classifiers, SVM considers a controllable confidence margin around its boarder which lead to both the minimizing the over-fitting with achieving acceptable results in the situation that small sample problem imposes to our experiment. As we can see in a similar research performed by Sabeti et al. (32), who employed LDA and Adaboost classifiers to assess a bigger population of controls and patients, while SVM enable us to take the same results with much less samples. This similarity to take similar results with different population indicates the capability of SVM in handing small data. In contrast, if we train LDA or Adaboost classifiers with much lower training samples (number of patients and controls); the performance of both classifiers would be remarkably declined because they do not consider any margin while the features are learnt.

In conclusion, ApEn is introduced as a powerful feature which is computed fast and acts precisely to extract informative information to classify psychotic disease from the controls.

## Authors' Contributions

MT designed a framework in which the project is defined, matched the achievement with the physiological basis of schizophrenic patients which published in the text book. RB suggested all of the methodologies in this paper, improved the first draft. MS implemented all programs and codes, produced the results and also provided the first draft of paper. AT performed the statistical analysis to prove the significance of the results. All authors read and approved the final manuscript.
